# Classification Strategies for P300-Based BCI-Spellers Adopting the Row Column Paradigm

**DOI:** 10.3390/s22239159

**Published:** 2022-11-25

**Authors:** Sofien Gannouni, Kais Belwafi, Nourah Alangari, Hatim AboAlsamh, Abdelfettah Belghith

**Affiliations:** 1 Department of Computer Science, College of Computer and Information Sciences, King Saud University, Riyadh 11543, Saudi Arabia; 2 Electrical Engineering and Computer Science Department, Khalifa University, Abu Dhabi P.O. Box 127788, United Arab Emirates

**Keywords:** brain–computer interface, P300 spellers, P300 row/column paradigm, n-class classification, ensemble classifiers

## Abstract

Acknowledging the importance of the ability to communicate with other people, the researcher community has developed a series of BCI-spellers, with the goal of regaining communication and interaction capabilities with the environment for people with disabilities. In order to bridge the gap in the digital divide between the disabled and the non-disabled people, we believe that the development of efficient signal processing algorithms and strategies will go a long way towards achieving novel assistive technologies using new human–computer interfaces. In this paper, we present various classification strategies that would be adopted by P300 spellers adopting the row/column paradigm. The presented strategies have obtained high accuracy rates compared with existent similar research works.

## 1. Introduction

Severe motor disabilities strongly threaten patients’ quality of life by deteriorating their ability to communicate with other people and making them lose their independence. The brain–computer interface (BCI) has emerged over the past few decades as the most important technology that aims to assist severely disabled people in regular everyday activities by offering them an efficient and easy-to-use muscle-independent pathway to control, communicate and interact with their environment [1]. BCI systems acquire and measure brain activity and translate it into control commands. Electroencephalography (EEG) is one of the most known non-invasive methods that is used by BCI systems to measure cerebral activity.

BCI-spellers were the first BCI applications that enabled people with severe motor disabilities to regain the ability to communicate with their environment. Various BCI-spellers have been proposed over the past few decades [2]. BCI-spellers are usually controlled either by motor imagery (MI) [3,4,5,6] or by event-related potentials (ERP) [2]. An MI-based BCI-speller derives its output from brain activity that is directly and consciously controlled by the user, independently from external events, by focusing on a specific mental task, i.e., imagining the movement of a muscle. An ERP-based BCI-speller derives its output from brain activity changes arising in reaction to external stimuli. ERPs are defined as changes in EEG signals during or after presenting external events (stimuli). ERP-based BCI-spellers are categorized to P300 [7,8,9,10] or steady-state visual evoked potential (SSVEP) spellers [11,12,13,14,15,16]. A P300 ERP is a positive peak in EEG that is elicited by visual or auditory stimuli. It appears about 300 ms after the stimuli start. SSVEP is characterized by positive and negative deflection in EEG signals arising in response to a visual stimulus that is flickering at a specific constant frequency.

P300-based spellers have a relatively high information transfer rate and require minimal user training (compared to MI). Contrary to MI and SSVEP-based spellers, P300-based spellers enable BCI systems to spell a wide range of words and support a high number of commands. Unfortunately, the use of a limited number of imagined movements has considerably reduced the ability of MI-based spellers to support a wide range of symbols. Indeed, to the best of our knowledge, very few imagined movements such as left- or right-hand movement, foot movement, tongue movement and eye gazing have been considered by MI-based spellers in the literature. On the other hand, SSVEP-based spellers do not require subject training or system calibration. Unfortunately, it is very difficult to discriminate between SSVEP responses when the number of presented stimuli is increased. Indeed, in such a case, it is very difficult to discriminate between various flickering visual stimuli with close frequencies. Moreover, it has been observed that some people have low SSVEP responses which reduced drastically the accuracy of the BCI system to detect such responses. All these reasons made P300-based spellers the most commonly used spellers by BCI systems. This paper overviews P300 paradigms and P300-based spellers paradigms.

BCI-spellers usually correspond to graphical representations of letters, numbers, and symbols which are controlled using MI or ERPs for spelling and typing. Most of the research papers dealing with BCI-spellers focus mainly on the design and implementation of the Graphical User Interface (GUI) of the BCI-speller which is the front-end of the BCI system. However, more attention should be given to the back-end of the BCI system which consists of EEG signal processing algorithms to increase the performance of the BCI system. It is very attractive to design and develop strategies to boost signal processing algorithms. Some research studies are continuously searching to improve the accuracy of the P300 speller. For example, in [17], an adaptive channel selection method is proposed to enhance the classification accuracy of the P300 potential. It efficiently permits channel selection by introducing multiple kernel learning (MKL) to select the model, mapping the EEG signals in different acquisition channels into other feature spaces through different kernel functions. So, it constructs MKL by linear weighting and uses many training sessions to learn weight coefficients to select the optimal sampling set channel combination adaptively. Other research studies focus on the development of deep learning algorithms to maximize the prediction rate of the P300 potential. In [18], a capsule network algorithm called ERP-CapsNet is proposed to perform ERP detection in a BCI-speller application. The experimental results on BCI Competition datasets and the Akimpech dataset show that ERP-CapsNet achieves a classification accuracy of about 65% for three subjects.

The current work has been conducted within a funded research project which aims to promote the accessibility of people with severe disabilities by developing a series of integrated Brain controlled tools such as mail–client, Web browser and command–line interface [19]. The developed prototypes adopted a P300-based BCI-speller. In this regard, several strategies have been explored during the project to classify P300 responses and to predict users’ desired symbols and/or commands. This manuscript describes all classification strategies we have explored during this project including a novel classification strategy that adopted a new partitioning approach to spread trials of the training dataset over an ensemble of classifiers. The results of the novel strategy outperformed all previous approaches including the winner of the BCI competition. To the best of our knowledge, there is no previous work that has adopted the proposed partitioning approach and the proposed classification strategy. Moreover, this work shows how a multi-class classification problem could be solved using two-phase classification strategies.

The remainder of this paper is organized as follows: Section 2 describes the different P300 paradigms and highlights the most commonly used P300 speller paradigms. Section 3 introduces the different annotations used in this paper. Section 4 describes different classification strategies for P300 spellers. Section 5 presents the results obtained by the discussed classification strategies and compares these results with those obtained by similar research works. Section 6 summarizes the current research work.

## 2. P300 Paradigms

The P300 is arising in response to an external event that appears as a positive deflection in voltage at the brain’s parietal lobe. It can be measured 300 milliseconds after the stimulus starts [20]. Thus, to detect and record these deflections, the EEG electrodes should cover the brain’s parietal lobe. Many prototypes have adopted this paradigm [19,21,22,23,24,25,26,27,28]. They are all characterized by a high degree of accuracy and require a short time of training. In our review of the literature, we identify three main P300 paradigms. The first one is the single-stimulus paradigm which includes one type of stimuli [29]. The second one is called the oddball paradigm, where the system shows a random sequence of two types of stimuli ’the target stimulus’ that infrequently appears in a sequence of stimuli, and the ’non-target’ stimulus, which appears more frequently [30,31]. The user focuses on a specific stimulus, “target”, that represents the user’s desire. The target stimulus elicits a P300 response while the other stimuli do not [30]. The last paradigm is the three-stimulus paradigm which contains three types of stimulus: target, standard, and distracter (also referred to as probes or novels). In the three-stimulus paradigm, the novel stimuli are presented infrequently and produce a P300 response that is different from the typical P300, which represents the response to the target (P3b), so this “novelty” P300 is called the (P3a) [29].

### 2.1. P300 Speller Paradigms

Different P300 Speller paradigms have been proposed in the literature and have developed distinct protocols. We hereby describe the most widely known P300 paradigms.

### 2.2. Row/Column Paradigm (RC)

Farwell and Donchin introduced the P300 row/column paradigm by presenting for subjects a 6 × 6 character matrix [7]. The subject is invited to concentrate on the character he desires to spell. For the spelling of a single character, rows and columns of the matrix are periodically flashed in random order. A P300 response is elicited 300 ms after the row or the column that contains the character the user wants to spell is flashed. After analyzing the user’s brain activity after each flash, P300 responses are detected, and consequently, the target character is identified as the intersection of the column and row that have elicited P300 responses.

### 2.3. Single Character Paradigm (SC)

In this paradigm, only one character is flashed at a time. This paradigm has a low accuracy rate. Moreover, SC can be prone to the crowding effect [29].

### 2.4. Checkerboard Paradigm (CB)

A novel P300-based stimulus presentation paradigm is presented in [8] to handle the adjacency problems and double flash in RC. A matrix with dimension 8 × 9 containing 72 elements is defined. The matrix is superimposed on a checkerboard. The checkerboard is split into two matrices, each with a dimension of 6 × 6. The first matrix contains the white cells from the checkerboard, and the second contains the black cells. Before each flash sequence, the checkerboard items will randomly populate the two matrices. So, the user sees a random group of six items flashing. The checkerboard layout controls the adjacency-distraction error because the adjacent cells will not be included in the same group. The rows of the matrix flash first randomly, then the columns flash. Any item will not flash again, at least for six intervening flashes; this will eliminate the double flash problem and avoid overlapping target epochs.

### 2.5. Region-Based Paradigm (RB)

In the RB paradigm [29,32], the screen was partitioned into seven regions. With 49 symbols (26 alphabet, 10 numeric, and 13 special characters). The 49 symbols are distributed among seven regions that are flashing randomly. The user focuses on the region that contains the target, then the system detects the region that contains the target by detecting the P300 response. Next, the seven symbols in the target region are distributed among the seven regions. Again the seven regions flash and then the system will identify the target symbol. This method provides more input characters. Moreover, avoid crowding effect and adjacency problem.

### 2.6. Comparison between RC, SC, CB, RB Paradigms

The authors in [33] have compared SC with RC P300 paradigms and state that only 55.3% of the trained subjects can spell with 100% accuracy in SC, whereas 72% of these subjects can spell with 100% in RC. Another study [29] has conducted experiments on six subjects (males 20–25 years old). Each participant completed the test with the SC, RC, and RB P300 paradigms. The participants were asked to spell two words. The total accuracy was 95% for RB, 85% for RC, and 72% for SC. In [8], experiments have been conducted on 18 participants (11 men, 7 women—2 women, and one man with ALS). Each participant completed two sessions of the experiments, one for RC and a second for CB. The CB accuracy was 91.52%, whereas the RC accuracy was 77.34. Table 1 compares between the different P300 speller paradigms.

## 3. Terminology and Annotations

Let us consider a matrix of symbols, called commands, denoted *M*, of dimension n×m; *n* and *m* correspond to the numbers of rows and columns of the matrix *M*, respectively.

(1) $$M=\left[\begin{array}{ccccc} C_{1}^{1} & \cdots & C_{1}^{j} & \cdots & C_{1}^{m}\\ \vdots & \; & \vdots & \; & \vdots \\ C_{i}^{1} & \cdots & C_{i}^{j} & \cdots & C_{i}^{m}\\ \vdots & \; & \vdots & \; & \vdots \\ C_{n}^{1} & \cdots & C_{n}^{j} & \cdots & C_{n}^{m} \end{array}\right]$$

**Figure 1:**
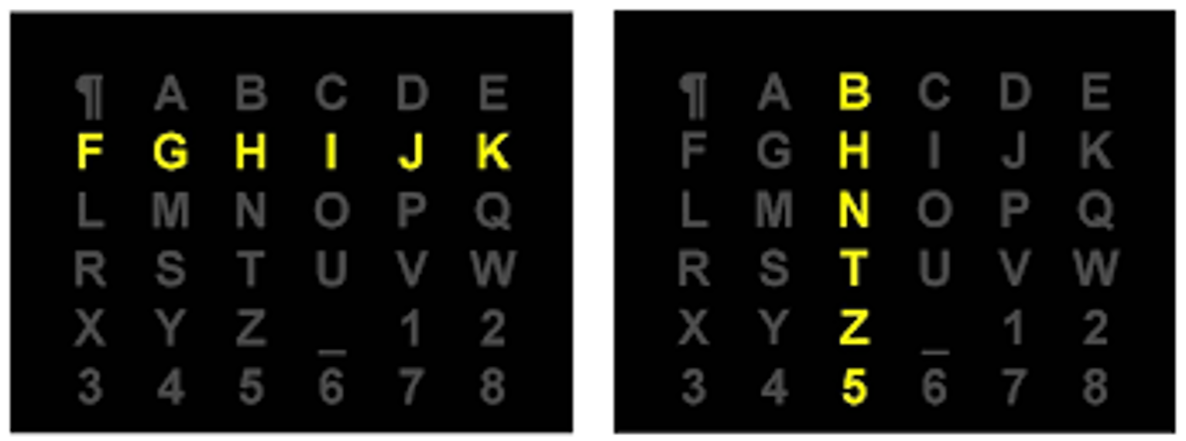
Intensification of the second row and the third column of a matrix of symbols.

The intensification denoted *I* is the process of flashing (intensifying the luminosity) all symbols of a given row or column of the matrix *M*. Figure 1 shows an illustration of the intensifications of the third column and of the second row of a matrix of symbols *M*, respectively. An intensification *I* elicits a single post-stimulus signal denoted ξ(I).

A sequence of intensification denoted by *S* represents an ordered collection of intensifications, where all rows and columns of *M* are intensified randomly once. Thus, *S* is formed by (n+m) distinct intensifications, denoted $S=<I_{1}^{i},I_{2}^{j},\cdots ,I_{n+m}^{k}>$. Each component *I* corresponds to an intensification of a row/column of *M*. In fact, $I_{i}^{j}$ means that the *i*th intensification of the sequence *S* occurs in the *j*th row/column of the matrix *M*. We notice that rows and columns of *M* are indexed using unique serial numbers, as shown in the following matrix (Equation (2)).

(2) $$\begin{array}{cccc} m & + & 1 & \Rightarrow \\ \vdots & & & \\ m & + & i & \Rightarrow \\ \vdots & & & \\ m & + & n & \Rightarrow \end{array}\begin{array}{c} \begin{array}{ccccc} 1 & \;\cdots \; & j & \;\cdots \; & m \end{array}\\ \left(\begin{array}{ccccc} ⇓ & & ⇓ & & ⇓\\ C_{1}^{1} & \cdots & C_{1}^{j} & \cdots & C_{1}^{m}\\ \vdots & & \vdots & & \vdots \\ C_{i}^{1} & \cdots & C_{i}^{j} & \cdots & C_{i}^{m}\\ \vdots & & \vdots & & \vdots \\ C_{n}^{1} & \cdots & C_{n}^{j} & \cdots & C_{n}^{m} \end{array}\right) \end{array}$$

A single sequence of intensification *S* elicits (n+m) post-stimulus signals denoted by ξ(S) corresponding to the following ordered collection of post-stimulus signals:

- $\xi \left(S\right)=<\xi \left(I_{1}^{i}\right),\xi \left(I_{2}^{j}\right),\cdots ,\xi \left(I_{n+m}^{k}\right)>$
- |ξ(S)|=(n+m)

For the selection, denoted by σ, of a single symbol/command $c_{i}^{j}$ of *M*, α sequences of intensifications are performed successively. As such, every row/column of *M* is intensified α times during the same selection σ but in random orders.

(3) $$\sigma =\overset{\alpha }{\underset{i=1}{⋃}}S_{i}$$

$S_{i}$ is the *i*th sequence of the selection σ. Thus, a selection σ of a single command elicits a set of post-stimulus signals denoted ξ(σ):

(4) $$\xi \left(\sigma \right)=\overset{\alpha }{\underset{i=1}{⋃}}\xi \left(S_{i}\right)=\overset{\alpha }{\underset{i=1}{⋃}}<\xi \left(I_{1,i}^{x}\right),\xi \left(I_{2,i}^{y}\right),\cdots ,\xi \left(I_{(n+m),i}^{z}\right)>$$

$I_{i,j}^{k}$ corresponds to the *i*th intensification of the *j*th sequence $S_{j}$ and which occurs in the *k*th row/column of the matrix of symbols *M*. ξ(σ) is composed of α×(n+m) post-stimulus signals.

(5) $$\left|\xi \left(\sigma \right)\right|=\sum_{i=1}^{\alpha }\left|\xi (S_{i})\right|=\sum_{i=1}^{\alpha }\left(n+m\right)=\alpha \times \left(n+m\right)$$

## 4. Classification Strategies

The classification problem addressed in this paper is a multi-class classification problem and it is solved in two steps. In the first step, a binary classification problem is addressed and it consists of predicting if an EEG post-stimulus signal corresponds to a P300 response or not. In the second phase, a multi-class classification problem is addressed and it consists of identifying the symbol that the user wants to spell.

Various design choices could be considered to develop a reliable and efficient solution. We describe hereafter the most relevant choices to build an efficient classification strategy:

- A single classifier or an ensemble of classifiers: A classifier is a machine learning model that allows us to identify, based on a set of attributes called features, which group, called a class, an object belongs to. Advances in machine learning have shown that an ensemble of classifiers effectively improves the classification accuracy of a single classifier. An ensemble of classifiers applies fusion techniques to combine predictions of single classifiers called base learners. However, the size of the training dataset is a critical issue in designing a classification strategy based on an ensemble of classifiers rather than on a single classifier. Indeed, the size of the training dataset impacts the performance of a machine-learning model. Large datasets lead to higher accuracy classification while small datasets degrade the system performance due to over-fitting.
- A heterogeneous or a homogeneous ensemble of classifiers: If the training dataset is large enough to adopt an ensemble of classifiers, the most important question that should be raised at that stage is: should we adopt distinct classifiers or distinct instances of the same classifier. Homogeneous ensembles develop the models of the base learners using the same classification algorithm. In heterogeneous ensembles, the base learners adopt distinct classification algorithms.
- Voting or stacking: In the case of an ensemble of classifiers, the final decision is obtained by applying a voting or a stacking fusion technique on the different labels predicted by the various base learners. Voting allows us to identify the class that has been predicted (voted) by the majority of base learners. Stacking uses the outputs of the base learners as features to train another classifier which is called a meta-learner, which will make the final decision.
- Replication or fragmentation of the training dataset: In the case of an ensemble of classifiers being adopted, there are two approaches to spread the training dataset over the base learners. Fragmentation allows us to decompose the training dataset into distinct fragments each of which will be used to train a single base learner. Replication allows us to train every base learner using the whole dataset. Replication is applicable only in case the base learners implement distinct classification algorithms, i.e., in the case of heterogeneous ensembles of classifiers.

**Figure 2:**
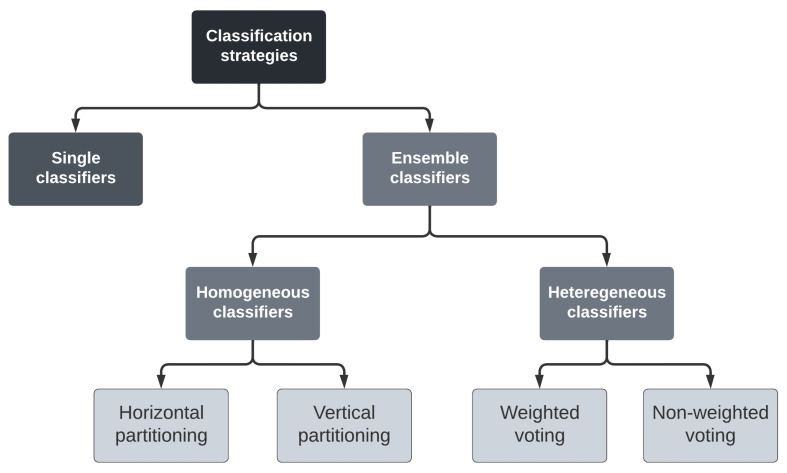
Classification strategies.

Figure 2 presents the different classification strategies investigated across this research work to predict a symbol from a matrix of symbols. These strategies are presented and discussed in the following sub-sections.

### 4.1. Single Classifier Strategy

The whole training dataset is used to train a 2-class classifier in the first phase. As such, the classifier is trained to predict if a post-stimulus EEG signal corresponds to a P300 response or not. For example, given a post-stimulus signal corresponding to an intensification $I_{i,j}^{k}$ of a given row or column of *M*, the prediction method denoted ρ returns a value ranging between 0 and 1, which corresponds to the probability that the signal ξ(I) is a P300 response or not.

(6) $$\rho \left(\xi \left(I_{i,j}^{k}\right)\right)=v\;such\;that\;v\in \left[0,1\right]$$

Given a sequence of intensifications $S_{i}$, the parsing method denoted τ returns a row vector, denoted by $U_{i}$, the values of which are obtained using the prediction method ρ.

(7) $$\tau \left(\xi \left(S_{i}\right)\right)=U_{i}$$

$U_{i}$ is a row vector composed of the elements <$u_{i}^{1},u_{i}^{2},\cdots ,u_{i}^{n+m}$> such that:

(8) $$u_{i}^{j}=\rho \left(\xi \left(I_{x,i}^{j}\right)\right)$$

$u_{i}^{j}$ is the probability that the intensification, whatever its order/rank, of the *j*th row/column of *M* that happens during the *i*th sequence of intensifications $S_{i}$ has elicited a P300 response or not. Thus, $\tau (\xi \left(S_{i}\right))$ is computed as follows:

(9) $$\tau \left(\xi \left(S_{i}\right)\right)=<\rho \left(\xi \left(I_{x,i}^{1}\right)\right),\rho \left(\xi \left(I_{y,i}^{2}\right)\right),\cdots ,\rho \left(\xi \left(I_{z,i}^{n+m}\right)\right)>$$

Every row and column of *M* is intensified once during the same sequence. Hence, the parsing method τ identifies which intensifications of a given sequence $S_{i}$ have elicited a P300 response and which have not. It will determine for every row/column of *M* whether its corresponding intensification that occurs during the given sequence $S_{i}$ has elicited a P300 response or not.

We remind that during the same selection σ, every row and column of *M* is intensified α times. So, given a selection σ of a single character, the corresponding α sequences of intensifications are processed sequentially by the parsing method τ leading to α row vectors each of which corresponds to $\tau {\left(\xi \left(S_{i}\right)\right)}_{1\leq i\leq \alpha }$. The α row vectors $U_{i}=\tau {\left(\xi \left(S_{i}\right)\right)}_{1\leq i\leq \alpha }$ are then used to average the probability that the intensifications of rows and columns of *M* happening during the selection σ have elicited P300 responses. These probabilities are computed using the following prediction function Ψ:

(10) $$\begin{array}{cc} \Psi (\xi (\sigma )) & =\frac{1}{\alpha }<\sum_{i=1}^{\alpha }\tau (\xi \left(S_{i}\right))>\;\\ \; & =\frac{1}{\alpha }<\sum_{i=1}^{\alpha }U_{i}>\;\\ \; & =\frac{1}{\alpha }<\sum_{i=1}^{\alpha }u_{i}^{1},\sum_{i=1}^{\alpha }u_{i}^{2},\cdots ,\sum_{i=1}^{\alpha }u_{i}^{n+m}>\;\\ \; & =\frac{1}{\alpha }<\sum_{i=1}^{\alpha }\rho (\xi \left(I_{x,i}^{1}\right)),\sum_{i=1}^{\alpha }\rho \left(\xi \left(I_{y,i}^{2}\right)\right),\cdots ,\sum_{i=1}^{\alpha }\rho (\xi \left(I_{z,i}^{n+m}\right))>\; \end{array}$$

As such Ψ(ξ(σ)) returns a row vector denoted by *V* as follows:

(11) $$\Psi \left(\xi \left(\sigma \right)\right)=<v^{1},v^{2},\cdots ,v^{m},v^{m+1},\cdots ,v^{n+m}>$$

such that

(12) $$v^{j}=\frac{1}{\alpha }\times \sum_{i=1}^{\alpha }\rho (\xi \left(I_{x,i}^{j}\right))$$

Given a selection σ, we can identify the user’s desired symbol by maximizing the results of the prediction function Ψ(ξ(σ)). We select the row *x* and the column *y* of *M*’ that have most probably elicited P300 responses. We notify that *y* corresponds to the column number maximizing the score $v^{y}$.

(13) $$v^{y}=Max_{i=1}^{m}\left(v^{j}\right)\;where\;v^{j}=\frac{1}{\alpha }\times \sum_{i=1}^{\alpha }\rho (\xi \left(I_{x,i}^{j}\right))$$

*x* is the number of the row that maximizes the score $v^{m+x}$.

(14) $$v^{m+x}=Max_{i=1}^{n}\left(v^{m+i}\right)$$

Thus, we consider that the symbol $c_{x}^{y}$ of *M* is most probably the user’s desired symbol.

### 4.2. Ensemble Classifiers Strategy

In the case of ensemble classifiers, two options are possible. The first option is called Heterogeneous Ensemble Classifiers, which consists of training different classifiers using the same training dataset. The second option is called Homogeneous Ensemble Classifiers, which consists of training different instances of the same classifier using different partitions of the training dataset.

Let us consider a training dataset, denoted *D*, comprising post-stimulus signals corresponding to the selection of β symbols (commands) $c_{i}^{j}$ of *M*.

(15) $$D=\overset{\beta }{\underset{j=1}{⋃}}\xi \left(\sigma^{j}\right)=\overset{\beta }{\underset{j=1}{⋃}}\overset{\alpha }{\underset{i=1}{⋃}}\xi \left(S_{i}^{j}\right)$$

where $S_{i}^{j}$ is the *i*th sequence $S_{i}$ that occurs during the *j*th selection. So, the dataset *D* consists of β×α×(n+m) post-stimulus training signals.

(16) $$\left|D\right|=\sum_{i=1}^{\beta }\left|\xi (\sigma_{i})\right|=\sum_{i=1}^{\beta }\alpha \times (n+m)=\beta \times \alpha \times \left(n+m\right)$$

#### 4.2.1. Heterogeneous Ensemble Classifiers Strategy

This step is often known as decision-level fusion, where different modalities are utilized for separate training models. An aggregation function is used at the end to determine the final decision by combining the different prediction results of distinct models. In such a case, the whole training dataset *D* is used to train *N* different 2-class classifiers, leading to an ensemble of distinct classifiers. Every classifier $C_{i:1\cdots N}$ is trained to predict if a signal contains a P300 response or not. Every classifier $C_{i:1\cdots N}$ builds its prediction model. Thus every classifier $C_{i:1\cdots N}$ will customize the prediction method ρ, denoted $\rho_{i:1\cdots N}$, according to its prediction model. The total number of trials used to train and build the model of every classifier is defined using the following expression:

(17) TotalnumberOftrials=β×α×(n+m)

There are two different approaches to building the final decision of this classification strategy: non-weighted voting and weighted voting.

a. Non Weighted Voting

In this case, the different classifiers play the same role to predict the selected symbol/command. Given a selection σ, the corresponding α sequences of intensifications are parsed simultaneously by the *N* distinct classifiers. Every classifier $C_{i:1\cdots N}$ calculates $\Psi_{i}\left(\xi \left(\sigma \right)\right)$ and returns a row vector denoted $V_{i}$ as follows:

(18) $$\Psi_{i}\left(\xi \left(\sigma \right)\right)=<v_{i}^{1},v_{i}^{2},\cdots ,v_{i}^{m},v_{i}^{m+1},\cdots ,v_{i}^{n+m}>$$

such that

(19) $$v_{i}^{j}=\frac{1}{\alpha }\times \sum_{k=1}^{\alpha }\rho_{i}\left(\xi \left(I_{x,k}^{j}\right)\right)$$

The results $V_{i}=\Psi_{i}{\left(\xi \left(\sigma \right)\right)}_{i:1\cdots N}$ obtained by the different classifiers $C_{i:1\cdots N}$ are then combined to calculate the global decision as follows:

(20) $$\Psi_{global}\left(\xi \left(\sigma \right)\right)=\frac{1}{N}\times \sum_{i=1}^{N}\Psi_{i}\left(\xi \left(\sigma \right)\right)$$

$\Psi_{global}\left(\xi \left(\sigma \right)\right)$ returns a row vector denoted $R=<r^{1},r^{2},\cdots ,r^{n+m}>$ such that

(21) $$r^{j}=\frac{1}{N}\times \sum_{i=1}^{N}\frac{1}{\alpha }\times \sum_{k=1}^{\alpha }\rho_{i}\left(\xi \left(I_{x,k}^{j}\right)\right)=\frac{1}{N}\times \frac{1}{\alpha }\times \sum_{i=1}^{N}\sum_{k=1}^{\alpha }\rho_{i}\left(\xi \left(I_{x,k}^{j}\right)\right)$$

$r^{j}$ is the probability that the intensification which occurs in the *j*th row/column of *M* has elicited a P300 response.

Given a selection σ, we can determine the user’s desired symbol by maximizing the results of the prediction function $\Psi_{global}\left(\xi \left(\sigma \right)\right)$. Using the values of the row vector $W=\Psi_{global}\left(\xi \left(\sigma \right)\right)$ we identify the column and the row that have most probably elicited P300 responses. Considering *y* is the number of the column of *M* that has most probably elicited P300 responses. Thus, *y* is the column number that maximizes the score $r^{y}$.

(22) $$r^{y}=Max_{i=1}^{m}\left(r^{j}\right)$$

Let *x* be the number of the row of *M* that has most probably elicited P300 responses. *x* is the number of the row that maximizes the score $r^{m+x}$.

(23) $$r^{m+x}=Max_{i=1}^{n}\left(r^{m+i}\right)$$

Thus, we consider that the symbol $c_{x}^{y}$ of *M* is the most probably user’s desired symbol.

b. Weighted Voting

In this case, we apply the same strategy as the non-weighted voting approach except that the decisions of the involved classifiers are waved to generate the global decision. Every classifier $C_{i:1\cdots N}$ is assigned a weight denoted $w_{i}$ that corresponds to the accuracy of $C_{i:1\cdots N}$ during the training phase. As such the results obtained by the different classifiers $C_{i:1\cdots N}$ are combined to predict the global decision using the following prediction function $\Psi_{global}\left(\xi \left(\sigma \right)\right)$ to calculate the global decision as follows:

(24) $$\Psi_{global}\left(\xi \left(\sigma \right)\right)=\frac{1}{N}\times \sum_{i=1}^{N}w_{i}\times \Psi_{i}\left(\xi \left(\sigma \right)\right)$$

$\Psi_{global}\left(\xi \left(\sigma \right)\right)$ returns a row vector denoted $R=<r^{1},r^{2},\cdots ,r^{n+m}>$ such that

(25) $$r^{j}=\frac{1}{N}\times \sum_{i=1}^{N}w_{i}\times \frac{1}{\alpha }\times \sum_{k=1}^{\alpha }\rho_{i}\left(\xi \left(I_{x,k}^{j}\right)\right)=\frac{1}{N}\times \frac{1}{\alpha }\times \sum_{i=1}^{N}w_{i}\times \sum_{k=1}^{\alpha }\rho_{i}\left(\xi \left(I_{x,k}^{j}\right)\right)$$

$r^{j}$ is the probability that the intensification that occurs in the *j*th row/column of *M* has elicited a P300 response.

Given a selection σ, we can determine the user’s desired symbol by maximizing the results of the prediction function $\Psi_{global}\left(\xi \left(\sigma \right)\right)$. Using the values of the row vector $R=\Psi_{global}\left(\xi \left(\sigma \right)\right)$ we identify the column and the row that have most probably elicited P300 responses. Let *y* and *x* be the numbers of the column and the row of *M* that has most probably elicited P300 responses, respectively. *y* and *x* are obtained by applying Equations (22) and (23), respectively.

Thus, we consider that the symbol $c_{x}^{y}$ of *M* is the most probably user’s desired symbol.

#### 4.2.2. Homogeneous Ensemble Classifiers Strategy

In this case, the training dataset *D* is split into disjoint partitions (portions). Every partition is used to train an instance of the same 2-class classifier, leading to an ensemble of homogeneous classifiers. Every classifier is as such trained to predict if a signal contains a P300 response or not. The training dataset *D* could be split into two different approaches: Vertical or Horizontal partitioning.

a. Horizontal Partitioning

We remind that the dataset contains β selections $\sigma_{i}$. Every selection $\sigma_{i}$ is composed of α sequences of intensifications denoted by $S_{i}^{j}$. $S_{i}^{j}$ represents the *j*th sequence of the *i*th selection.

In the horizontal partitioning strategy, the signals of the training dataset *D* are spread over α partitions denoted $\pi_{i:1\cdots \alpha }$ each of which is defined as follows:

(26) $$\pi_{i:1\cdots \alpha }=\overset{\beta }{\underset{j=1}{⋃}}\xi \left(S_{j}^{i}\right)$$

A partition $\pi_{i:1\cdots \alpha }$ contains all *i*th sequences of intensifications of the β selections. As such, the partition $\pi_{1}$ contains all first sequences of the β selections, the partition $\pi_{2}$ contains all second sequences of the β selections and so on. The obtained partitions have to satisfy the following properties:

(27) $$\overset{\alpha }{\underset{i=1}{⋃}}\pi_{i}=D\;\mathrm{and}\;\overset{\alpha }{\underset{i=1}{⋂}}\pi_{i}=⌀$$

Every partition $\pi_{i}$ is composed of β sequences of intensifications. Hence, a partition $\pi_{i}$ contains β×(n+m) post-stimulus signals.

(28) $$|\pi_{i}|=\sum_{j=1}^{\beta }\left|\xi (S_{j}^{i})\right|=\sum_{j=1}^{\beta }n+m=\beta \times \left(n+m\right)$$

Every partition $\pi_{i}$ is used to train a single 2-class classifier $C_{i:1\cdots \alpha }$, leading to an ensemble of α classifiers. Every classifier $C_{i:1\cdots \alpha }$ is as such trained to predict if a signal corresponds to a P300 response or not. Thus, the total number of trials used to train every classifier is defined using the following expression:

(29) TotalnumberOftrials=β×(n+m)

Given a selection σ, the corresponding α sequences of intensifications are parsed simultaneously by the α classifiers. Every classifier processes a single sequence of the α sequences of σ. Thus, a classifier $C_{i}$ will parse the $i_{th}$ sequence $S_{i}$ of σ. Every classifier $C_{i}$ calculates $\tau_{i}\left(\xi \left(S_{i}\right)\right)$ as follows:

(30) $$\tau_{i}\left(\xi \left(S_{i}\right)\right)=<\rho_{i}\left(\xi \left(I_{x,i}^{1}\right)\right),\rho \left(\xi \left(I_{y,i}^{2}\right)\right),\cdots ,\rho \left(\xi \left(I_{z,i}^{n+m}\right)\right)>$$

Every classifier $C_{i}$ returns a row vector denoted $V_{i}$ whose values are calculated as follows:

(31) $$v_{i}^{j}=\rho_{i}\left(\xi \left(I_{x,i}^{j}\right)\right)$$

$v_{i}^{j}$ is the probability that the intensification, whatever its order/rank, of the *j*th row/column of *M* that happens during the *i*th sequence of intensifications $S_{i}$ has elicited a P300 response or not. The different results (row vectors) $V_{i}=\tau_{i}{\left(\xi \left(S_{i}\right)\right)}_{i:1\cdots \alpha }$ calculated by the different classifiers $C_{i:1\cdots \alpha }$ are then combined to calculate the global decision as follows:

(32) $$\Psi_{global}\left(\xi \left(\sigma \right)\right)=\frac{1}{\alpha }\times \sum_{i=1}^{\alpha }\tau_{i}\left(\xi \left(\left(S_{i}\right)\right)\right)$$

$\Psi_{global}\left(\xi \left(\sigma \right)\right)$ returns a row vector denoted $R=<r^{1},r^{2},\cdots ,r^{n+m}>$ such that

(33) $$r^{j}=\frac{1}{\alpha }\times \sum_{i=1}^{\alpha }\rho_{i}\left(\xi \left(I_{x,i}^{j}\right)\right)$$

$r^{j}$ is the probability that the intensifications that occur in the *j*th row/column of *M* have elicited P300 responses.

Given a selection σ, we can identify the column and the row that have most probably elicited P300 responses by maximizing the results of the prediction function $\Psi_{global}\left(\xi \left(\sigma \right)\right)$. Let *y* and *x* be the numbers of the column and the row of *M* that has most probably elicited P300 responses, respectively. *y* and *x* are obtained by applying Equations (22) and (23), respectively. Thus, we consider that the symbol $c_{x}^{y}$ of *M* is the most probable user’s desired symbol.

b. Vertical Partitioning

In the vertical partitioning strategy, the selections are spread over a set of classifiers. So, we defined a collection composed of *N* different instances of the same classifier. Then, we split equally the β selections over the different instances $C_{i:1\cdots N}$. Thus, every classifier $C_{i:1\cdots N}$ will be trained on a subset of the training dataset composed of $\frac{\beta }{N}$ selections. As such, every instance $C_{i:1\cdots N}$ of the classifiers will be assigned a partition defined as follows:

(34) $$\pi_{i:1\cdots N}=\overset{i\times \frac{\beta }{N}}{\underset{j=\left(i-1\right)\times \frac{\beta }{N}+1}{⋃}}\xi \left(\sigma_{j}\right)$$

A partition $\pi_{i:1\cdots N}$ contains $\frac{\beta }{N}$ successive selections and satisfies the properties described by expression (33).

Every partition $\pi_{i}$ contains $\frac{\beta }{N}\times \alpha \times \left(n+m\right)$ post-stimulus signals.

(35) $$|\pi_{i}|=\sum_{j=\left(i-1\right)\times \frac{\beta }{N}+1}^{i\times \frac{\beta }{N}}\left|\xi (\sigma_{j})\right|=\sum_{j=\left(i-1\right)\times \frac{\beta }{N}+1}^{i\times \frac{\beta }{N}}\sum_{k=1}^{\alpha }\left|\xi (S_{j}^{k})\right|=\frac{\beta }{N}\times \alpha \times \left(n+m\right)$$

Every partition $\pi_{i}$ is used to train a 2-class classifier $C_{i:1\cdots N}$, leading to an ensemble of *N* classifiers. Every classifier $C_{i:1\cdots N}$ is as such trained to predict if a signal contains a P300 response or not. Thus, the total number of trials used to train every classifier is defined using the following expression:

(36) $$Total\;number\;Of\;trials\;=\frac{\beta }{N}\times \;\alpha \times \left(n+m\right)$$

Given a selection σ, the corresponding α sequences of intensifications are parsed simultaneously by the *N* classifiers. Every classifier $C_{i:1\cdots N}$ calculates $\Psi_{i}\left(\xi \left(\sigma \right)\right)$ and returns a row vector denoted $V_{i}$ (Equation (18)) whose values are calculated using Equation (19).

The different results (row vectors) $V_{i}=\Psi_{i}{\left(\xi \left(\sigma \right)\right)}_{i:1\cdots N}$ calculated by the different classifiers $C_{i:1\cdots N}$ are then combined to calculate the global decision as follows:

(37) $$\Psi_{global}\left(\xi \left(\sigma \right)\right)=\frac{1}{N}\times \sum_{i=1}^{N}\Psi_{i}\left(\xi \left(\sigma \right)\right)$$

$\Psi_{global}\left(\xi \left(\sigma \right)\right)$ returns a row vector denoted $R=<r^{1},r^{2},\cdots ,r^{n+m}>$ such that

(38) $$r^{j}=\frac{1}{N}\times \sum_{i=1}^{N}\frac{1}{\alpha }\times \sum_{k=1}^{\alpha }\rho_{i}\left(\xi \left(I_{x,k}^{j}\right)\right)=\frac{1}{N}\times \frac{1}{\alpha }\times \sum_{i=1}^{N}\sum_{k=1}^{\alpha }\rho_{i}\left(\xi \left(I_{x,k}^{j}\right)\right)$$

$r^{j}$ is the probability that the intensification that occurs in the *j*th row/column of *M* has elicited a P300 response.

Given a selection σ, we can identify the column and the row that have most probably elicited P300 responses by maximizing the results of the prediction function $\Psi_{global}\left(\xi \left(\sigma \right)\right)$. Let *y* and *x* be the numbers of the column and the row of *M* that has most probably elicited P300 responses, respectively. *y* and *x* are obtained by applying Equations (22) and (23), respectively. Thus, we consider that the symbol $c_{x}^{y}$ of *M* is the most probable user’s desired symbol.

## 5. Results and Discussion

### 5.1. Description of the Testing Dataset

A dataset provided by BCI competition, in [34], is used to test and check the effectiveness of the proposed approach and for comparison purposes. It contains the EEG signals of two subjects trying to select 85 characters (85 selections) during five different sessions. Each selection is recorded from the brain using 64 channels, most of which are put in the brain’s parietal lobe. Every character spelling corresponds to 180 = 12 × 15 post-stimulus labeled signals. So, each subject’s dataset is composed of 15,300 post-stimulus in total.

The testing dataset is composed of 100 characters spelling, equivalent to 18,000 = 180 × 100 post-stimulus signals. The diverse parameters of the selected dataset using the previous approach are summarized in Table 2.

Performing a benchmark test with only two subjects is insufficient in general. However, every subject was invited to select 100 characters which correspond to 18,000 trials (EEG signals) per subject. The number of trials is enough to make a significant benchmark test.

### 5.2. EEG Signals Pre-Processing and Features Extraction

We are interested in this work only in EEG signals that appeared after each intensification. As mentioned above, the evoked potentials appear 300 ms after the stimulus. Thus, samples between 0 to 667 ms posterior to the beginning of intensification are extracted for every channel. The sampling rate of the EEG signals was set to 240 Hz. To the best of our knowledge, this epoch is large enough to acquire efficient time features. The selected epoch is bandpass filtered using an infinite impulse response (IIR) filter. The order of the filter is set to eight as it is sufficient to keep the spectrum components between 0.1 and 10 Hz. Afterward, the filtered signals were decimated according to the high cut-off frequency, 10 Hz. The decimation process reduces the sampling frequency of a signal to a lower sampling frequency that differs from the original frequency. Decimation is also known as down-sampling. So, the extracted signal comprises only 14 samples at each electrode. Finally, each post-stimulus signal is transformed as a single vector composed of 896 = 14 × 64 samples. As mentioned above, the EEG signal has been filtered with an 8-order bandpass filter the cut-off frequencies of which are 0.1 and 10 Hz and has been decimated according to the high cut-off frequency. The decimation process reduces the sampling frequency of a signal to a lower sampling frequency that differs from the original frequency. Decimation also is known as down-sampling. Therefore, the extracted signal from a single channel is composed of 14 samples.

### 5.3. Results

The various strategies analyzed in this work have been evaluated using the dataset mentioned above. For every classification strategy, Table 3 shows the total number of psst-stimulus signals used for training the classifiers.

The benchmark dataset contains two subsets of trials. The first subset is composed of 85 selections per subject which correspond to 15,300 post-stimulus signals. This subset is used to train the classifier(s). Table 3 summarizes how these trials are spread over the classifiers according to the classification strategy. The second subset is composed, as mentioned in Table 2, of 100 selections per subject which correspond to 18,000 post-stimulus signals per subject too. The second subset is used for testing the accuracy of the classification strategies.

Given a selection σ, the α sequences of intensifications are parsed by the classifier(s) of a given strategy. The distinct (n+m) intensifications of every sequence are processed separately by the classifier(s) to determine which ones correspond to a P300 response and which do not. For every sequence of intensifications, the classifier(s) aggregates the (n+m) predictions and returns a row vector. Every strategy consolidates the distinct α row vectors, using appropriate formulas, to identify which character corresponds most probably to the selection σ.

Various classifiers are applied during the first phase of the classification problem to analyze and select the appropriate one for our application. For example, we have evaluated the linear discriminant analysis (LDA) [35], partial least squares regression (PLS) [36], logistic regression (REG) [37], and support vector machine (SVM) [19]. These algorithms were evaluated using the BCI competition dataset containing the recording of two subjects, A and B. Every classifier has been trained using 15,300 post-stimulus signals and tested using 18,000 post-stimulus signals. Table 4 summarizes the classification accuracy of each classifier, which represents the number of trials classified correctly. The maximum classification accuracy was reached using the SVM algorithm, where for subject A the accuracy achieved was 96% and 93% for subject B, respectively. Thus, the average classification accuracy using this technique is about 94.5%. Regarding subject A, the classification accuracy achieved was 93%, 94%, and 94% using LDA, PLS, and REG, respectively. The SVM fails to maintain high accuracy for subject B, where the highest accuracy was obtained using the PLS and REG classifiers.

SVM, LDA, PLS and REG classifiers have been fused to evaluate the performance of the ensemble heterogeneous classification strategy. Table 5 shows the accuracy rate of the ensemble classifiers for subjects A and B. The ensemble classifiers have been trained using 15,300 post-stimulus signals and have been tested using 18,000 post-stimulus signals. Using the non-weighted voting technique, the ensemble classifiers reach an accuracy rate of 88% and 91% for subjects A and B, respectively. The weighted voting techniques enhance the accuracy of the ensemble classifiers by achieving an accuracy rate of 89% for subject A. For subject B, the performance remains the same.

The ensemble homogeneous classification strategy was evaluated by fusing the predictions of different instances of the SVM classifier. In the case of vertical partitioning, we decided to split the training dataset into partitions each of which was composed of post-stimulus signals of five successive selections, leading to 17 different partitions. As such, every partition corresponded to 900 post-stimulus signals. Every partition has been used to train a single instance of the SVM classifier. Table 6 shows the accuracy rate of the ensemble classifiers for subjects A and B. The accuracy rate of the ensemble classifiers using the vertical partitioning reached an accuracy rate of 96% and 93% for subjects A and B, respectively.

In the case of horizontal partitioning, the training dataset was split into 15 partitions each of which was composed of post-stimulus signals that correspond to sequences that happen in the same order during the selections. Partition 1 contained post-stimulus signals of all first sequences. Partition 2 contained post-stimulus signals corresponding to all second sequences, etc. As such, every partition corresponded to 1020 post-stimulus signals. Every partition has been used to train a single instance of the SVM classifier. As shown in Table 6, the accuracy rate of the ensemble classifiers using the horizontal partitioning reached an accuracy rate of 98% and 96% for subjects A and B, respectively.

### 5.4. Benchmarks

For comparison purposes, Table 7 summarizes the accuracy rates obtained by the winners of the BCI competition.

Unexpectedly, the efficiency of the ensemble heterogeneous classification strategy is low compared to those of the single classification strategy. However, results show that, using a weighted voting technique or not, the ensemble heterogeneous classification strategy has obtained better accuracy rates than the third-ranked algorithm of the BCI competition. In the meantime, the proposed single classification strategy outperformed the second and third-ranked algorithms.

Compared to the winner, the proposed ensemble homogeneous classification strategies have obtained competitive results. Adopting the vertical partitioning approach, the ensemble homogeneous classification strategy has reached the same accuracy as the winner for subject A, but the accuracy has decreased by 2% for subject B. Using the horizontal partitioning, the ensemble homogeneous classification strategy outperformed the winner for both subjects A and B.

## 6. Conclusions and Future Work

P300 spellers offered serious opportunities for people with severe motor disabilities to interact efficiently with their environment. Different P300 speller paradigms have been proposed in the literature. This paper overviewed the most known P300 speller paradigms the Row/Column P300 speller paradigm still remains the most popular paradigm adopted by P300 spellers. This paper discussed various efficient classification strategies for the row/column P300 speller paradigm. These strategies were tested using a public dataset. The results were competitive compared to those obtained by the winner algorithms of the BCI competition using the same dataset. One of the presented strategies outperformed the winner of the competition.

The classification problem addressed in this paper was complex and it was solved in two steps. The first step aimed to predict if a post-stimulus EEG signal corresponded to a P300 response or not. Thus, this first step was a 2-class classification problem-solving. The second phase dealt with a multi-class classification problem since it aimed to predict the user’s desired symbol/command from a matrix of symbols.

Various strategies were discussed in this paper including single classifier strategy and ensemble classification strategies. The single classifier strategy was tested using four different 2-class classifiers: LDA, PLS, REG, and SVM. The average accuracy rate of such a strategy ranged between 92% and 96%. These distinct classifiers were fused to validate the ensemble heterogeneous classification strategy by applying a weighted voting and a non-weighted voting algorithm. Contrary to all expectations, the performance decreased compared to the single classification strategy. This lack of performance was fixed by the ensemble homogeneous classification strategy by fusing different instances of the same classifier, in this case, the SVM classifier. The ensemble was built based on how the dataset was split among the different instances. Two approaches were tested: horizontal and vertical partitioning. The ensemble homogeneous classification strategy using the vertical partitioning technique obtained results too close to the winner algorithm of the BCI competition. However, the ensemble classification strategy using the horizontal partitioning technique outperformed the winner algorithm of the competition. The average accuracy rate was improved by 2%.

In the future, we intend to extend our classification strategies to include deep learning algorithms and study their efficiency. Moreover, we are planning to combine the two selective attention methods P300 and SSVEP, or P300 with ERS/ERD motor imagery BCI mental strategies knowing that recent research showed that the subject can simultaneously produce both SSVEP and ERS/ERD.

## Figures and Tables

**Table 1:** Comparison between the P300 Speller Paradigms.

Paradigm	RC	SC	CB	RB
**Accuracy**	**Medium**	**Low**	**Very High**	**High**
Adjacency problem	✓	✓	✘	✘
Crowding Effect	✓	✓	✓	✘
Double flash	✓	✘	✘	✘

✓ Xexist ✘ not exist.

**Table 2:** Description of the benchmarking dataset.

	Notation	Formula	Value
The dimensions of the command Matrix *M*	n,m		6, 6
The number of post-stimulus signals during a single sequence of intensifications *S*.	|ζ(S)|	|ζ(S)|=n+m	12
The number of sequences of intensifications *S* in a single selection σ.	α		15
Total number of post-stimulus signals during a single selection.	|ζ(σ)|	|ζ(S)|=α×(n+m)	180
The number of selections (per subject) of the training dataset.	β		85
Total number of post-stimulus signals (per subject) of the training dataset.	|D|	|D|=β×α×(n+m)	15,300
The number of selections (per subject) of the testing dataset.	$\beta^{\prime }$		100
Total number of post-stimulus signals (per subject) of the testing dataset.	$|D^{\prime }|$	$|D^{\prime }|=\beta^{\prime }\alpha \left(n+m\right)$	18,000

**Table 3:** Settings of the different classification strategies.

	Number of	Total Number of Post-Stimulus Signals per Classifier	
	**Classifiers**	**Formula**	**Value**
Ensemble Heterogeneous Classifiers Strategy	4	|D|=β×α×(n+m)	15,300
Ensemble Homogeneous Classification Strategy with Vertical Partitioning	17	$\left|D\right|=\frac{\beta }{N}\times \alpha \times \left(n+m\right)$	900
Ensemble Homogeneous Classification Strategy with Horizontal Partitioning	15	|D|=β×(n+m)	1020

**Table 4:** Average accuracy of the single classification strategy.

Subject	LDA	SVM	PLS	REG
Subject A	93	96	94	94
Subject B	92	93	94	94

**Table 5:** Average accuracy of the ensemble heterogeneous classification strategy.

	Accuracy (%)	
**Subject**	**Weighted Voting**	**Non-Weighted Voting**
Subject A	89	88
Subject B	91	91

**Table 6:** Average accuracy of the ensemble homogeneous classification strategy using vertical or horizontal partitioning.

	Accuracy (%)	
**Subject**	**Horizontal Partitioning**	**Vertical Partitioning**
Subject A	98	96
Subject B	96	93

**Table 7:** Accuracy rates obtained by the winners of the BCI competition (%).

	Winners of the BCI Competition		
**Subject**	**1st**	**2nd**	**3rd**
Subject A	96	90.5	80
Subject B	95	90.5	80

## Data Availability

Not applicable.
